# An aldo-keto reductase with 2-keto-l-gulonate reductase activity functions in l-tartaric acid biosynthesis from vitamin C in *Vitis vinifera*

**DOI:** 10.1074/jbc.RA119.010196

**Published:** 2019-09-04

**Authors:** Yong Jia, Crista A. Burbidge, Crystal Sweetman, Emi Schutz, Kathy Soole, Colin Jenkins, Robert D. Hancock, John B. Bruning, Christopher M. Ford

**Affiliations:** ‡ Waite Research Institute, School of Agriculture, Food, and Wine, University of Adelaide, Adelaide 5064, Australia; § College of Science and Engineering, Flinders University, GPO Box 2100, Adelaide 5001, Australia; ¶ Cell and Molecular Sciences, James Hutton Institute, Invergowrie, Dundee DD2 5DA, United Kingdom; ‖ Institute of Photonics and Advanced Sensing, School of Biological Sciences, University of Adelaide, Adelaide 5005, Australia

**Keywords:** plant biochemistry, X-ray crystallography, structural biology, enzyme kinetics, enzyme mechanism, grapevine, tartaric acid synthesis, ascorbic acid, Vitis vinifera, 2-keto-L-gulonic acid, aldo-keto reductase, protein crystallization, substrate specificity, docking, D-isomer-specific 2-hydroxyacid dehydrogenase

## Abstract

Tartaric acid has high economic value as an antioxidant and flavorant in food and wine industries. l-Tartaric acid biosynthesis in wine grape (*Vitis vinifera*) uses ascorbic acid (vitamin C) as precursor, representing an unusual metabolic fate for ascorbic acid degradation. Reduction of the ascorbate breakdown product 2-keto-l-gulonic acid to l-idonic acid constitutes a critical step in this l-tartaric acid biosynthetic pathway. However, the underlying enzymatic mechanisms remain obscure. Here, we identified a *V. vinifera* aldo-keto reductase, Vv2KGR, with 2-keto-l-gulonic acid reductase activity. Vv2KGR belongs to the d-isomer–specific 2-hydroxyacid dehydrogenase superfamily and displayed the highest similarity to the hydroxyl pyruvate reductase isoform 2 in *Arabidopsis thaliana*. Enzymatic analyses revealed that Vv2KGR efficiently reduces 2-keto-l-gulonic acid to l-idonic acid and uses NADPH as preferred coenzyme. Moreover, Vv2KGR exhibited broad substrate specificity toward glyoxylate, pyruvate, and hydroxypyruvate, having the highest catalytic efficiency for glyoxylate. We further determined the X-ray crystal structure of Vv2KGR at 1.58 Å resolution. Comparison of the Vv2KGR structure with those of d-isomer–specific 2-hydroxyacid dehydrogenases from animals and microorganisms revealed several unique structural features of this plant hydroxyl pyruvate reductase. Substrate structural analysis indicated that Vv2KGR uses two modes (A and B) to bind different substrates. 2-Keto-l-gulonic acid displayed the lowest predicted free-energy binding to Vv2KGR among all docked substrates. Hence, we propose that Vv2KGR functions in l-tartaric acid biosynthesis. To the best of our knowledge, this is the first report of a d-isomer–specific 2-hydroxyacid dehydrogenase that reduces 2-keto-l-gulonic acid to l-idonic acid in plants.

## Introduction

l-Tartaric acid (TA)^3^ is a four-carbon organic acid rapidly synthesized in the early stage of grape (*Vitis vinifera*) berry development, which remains metabolically stable during berry ripening and the subsequent winemaking process (1, 2). TA confers a low pH and a “sharp” flavor to wine, affecting many quality aspects, such as color, taste, microbial stability, and aging potential. In addition, exogenous TA is widely used as a flavorant and an antioxidant additive in the food and wine industries. Earlier studies showed that the biosynthesis of TA in grapevine employs l-ascorbic acid (Asc) as its biological precursor (3–5), which thus attracts increasing research interest to this metabolic pathway (6–8).

Asc is a ubiquitous antioxidant present in the various tissues of plants (9). Asc plays critical roles in many aspects of plant development. It functions in a wide range of metabolic and physiological processes, such as photosynthesis (10), hormone biosynthesis (11), stress response (12), cell growth (9), and flowering time (13). Humans lack the ability to synthesize Asc and therefore depend on plant-based foods as the principle source of vitamin C (14). An understanding of how plants maintain Asc at the right level is still emerging (15); however, it is clear that Asc homeostasis involves a balance between biosynthesis and degradation. Multiple biosynthetic pathways and the corresponding molecular mechanisms have been well-characterized for Asc in plant cells (reviewed in Refs. 16 and 17). In addition, the molecular basis for the oxidation and recycling of Asc has also been revealed (17). However, limited attention has been drawn toward the metabolic degradation of Asc in plants, and the underlying genetic basis remains largely elusive.

**Figure 1.**
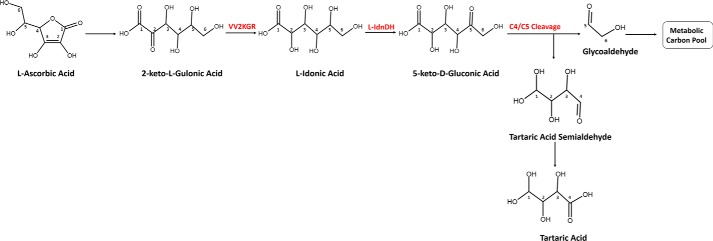
**TA biosynthetic pathway from the C4/C5 cleavage of Asc.** The carbon atoms in each molecule were indexed according to the numbering in Asc. VvL-IdnDH catalyzing the conversion of l-idonic acid to 5-keto-d-gluconic acid was characterized previously by DeBolt *et al.* (3). Vv2KGR responsible for the conversion of 2-keto-l-gulonic acid to l-idonic acid was identified in the present study.

Earlier studies provided preliminary insights into the breakdown process of Asc in plants. It was reported that Asc content in plant tissues is maintained at an appreciable turnover rate, ranging from 2–3% of the total pool per hour in barley and potato leaves (12, 18) and 0.5–4% in developing blackcurrant fruit (19) to about 13% in pea embryonic axes (20). Asc readily reacts with reactive oxygen species, resulting in the formation of monodehydroascorbate. Monodehydroascorbate is unstable and, if not rapidly reduced, disproportionates to Asc and dehydroascorbate (DHA) (16). It was suggested that the degradation of Asc mainly proceeds via the breakdown of DHA (20). DHA has an unstable lactone ring that can be hydrolyzed to 2,3-diketogulonate in aqueous solution. 2,3-Diketogulonate further degrades to various carbonate products, including CO_2_, oxalate, glyoxalate, glycerate, and threonate (21). Two enzymatic pathways of Asc degradation were proposed in plants that accumulate oxalate and tartrate (17, 22). The first pathway (Fig. 1) occurs in grapevine and some other *Vitaceae* plants that accumulate high levels of TA. In this pathway, the six-carbon Asc is cleaved at C4/C5, yielding TA and a two-carbon compound (4, 23, 24). The two-carbon fragment is recycled into the pentose phosphate pathway (24). The second pathway exists in some *Geraniaceae* plants, in which Asc is cleaved at C2/C3, producing oxalic acid and l-threonic acid (22, 25). The four-carbon l-threonic acid either enters the carbohydrate metabolic pool or is oxidized to TA (25). This pathway contributes significantly to the production of oxalic acid and may be present in many plants.

Earlier radioisotope tracer studies showed that Asc or DHA fed to grapevine is first converted to 2-keto-l-gulonic acid (2KLG), which is then reduced to l-idonic acid (IA). IA is oxidized to 5-keto-d-gluconic acid (4, 5). The six-carbon 5-keto-d-gluconic acid is cleaved at C4/C5, yielding the four-carbon l-*threo*-tetruronate and a two-carbon fragment (4, 5, 23). l-*threo*-Tetruronate is then oxidized to TA while the two-carbon fragment, possibly glycoaldehyde, is recycled into triose and hexose phosphate metabolism (23, 24). In this pathway, the conversion of IA to 5-keto-d-gluconic acid has been proposed as the rate-limiting step (5). The underlying gene responsible for this reaction has been identified as *VvL-IdnDH* in grapevine, which encodes an enzyme named l-idonate dehydrogenase that is highly homologous to sorbitol dehydrogenase (3). *VvL-IdnDH* represents a functional divergence from plant sorbitol dehydrogenase and is only conserved in some species (7). It is so far the only gene identified in this Asc degradation pathway.

In addition to the above-mentioned enzymatic reactions, the conversion of 2KLG to IA has also been suggested to be enzyme-catalyzed and reversible (4). However, no candidate enzyme controlling this putative enzymatic reaction has been identified to date. Interestingly, enzymes with the ability to catalyze the same chemical reaction have been reported in a number of microorganisms, including *Erwinia herbicola* (26), *Brevibacterium ketosoreductum* (27), *Escherichia coli* (28), and various acetic acid bacteria (29). In these microbes, 2KGRs are able to catalyze the reduction of 2,5-diketo-d-gluconate to 5-keto-d-gluconate, 2-keto-d-gluconate to d-gluconate, and 2KLG to IA, playing a role in the ketogluconate metabolic pathway (26–28). This class of enzyme belongs to the d-isomer–specific 2-hydroxyacid dehydrogenase (2KDH) superfamily, which catalyzes the reversible reduction of 2-oxoacids to the d-isomers of the respective 2-hydroxyacids. It should be noted that IA has a dextrorotatory (*d*) C2, although it is named as “l-idonic acid.” Most recently, a similar 2-keto-l-gulonate reductase, catalyzing the reversible reduction of 2KLG to IA, was identified from the bacteria *Ketogulonicigenium vulgare* (30) and the filamentous fungus *Aspergillus niger* (31), suggesting that this enzyme may be ubiquitous in all living microorganisms. However, no such enzyme has been reported in plants.

In this study, we report the identification of an aldo-keto reductase with 2-keto-l-gulonate reductase (Vv2KGR) from grapevine. Vv2KGR is able to catalyze the reversible reduction of 2KLG to IA, with NADPH as the preferred cofactor. We further determined the X-ray crystal structure of Vv2KGR to 1.58 Å resolution. Comparative structural analyses showed that Vv2KGR has a typical 2KDH fold and shares the highest structural similarity with plant hydroxyl pyruvate reductase (HPR). The substrate binding and catalytic mechanism were investigated in-depth by molecule docking. Our study is the first to report that a 2KDH can convert 2KLG to IA in plants.

## Results and discussion

### Identification and cloning of candidate gene

To identify the candidate enzyme catalyzing the conversion of 2KLG to IA (Fig. 1) in grapevine, the previously characterized *E. coli* 2-ketoaldonate reductase (yiaE, UniProt number P37666) (28) was used for homology searches. A consensus sequence (accession number TC59682) encoding a putative enzyme highly homologous (∼39% identity) to *E. coli* yiaE was identified by tBLASTn against the *V. vinifera* EST database. TC59682 was selected also based on its presence in EST libraries prepared from young grape berry, where TA is rapidly synthesized. The deduced amino acid sequence (313 amino acids, UniProt number A5CAL1) from TC59682 is named as *V. vinifera* 2-keto-l-gulonate reductase (Vv2KGR) in the present study. *E. coli* yiaE catalyzes the conversion of 2KLG to IA in the gluconic acid metabolism pathway. Considering the remote distance between *V. vinifera* and *E. coli*, the amino acid identity (∼39%) between Vv2KGR and yiaE is very significant, indicating that Vv2KGR may be able to convert 2KLG to IA as well. The full-length coding domain sequence of *Vv2KGR* was successfully amplified by PCR from a cDNA library of *V. vinifera* cv. Shiraz pre-véraison berry. Primary protein structural analyses showed that Vv2KGR and yiaE belong to the same 2KDH superfamily, containing typical 2KDH catalytic domain and NAD(P)H-binding domain signatures (Fig. S1). In plants, Vv2KGR displayed the highest sequence similarity with *Arabidopsis thaliana* HPR isoform 2 (AtHPR2, ∼75%) (32) while sharing 48 and 34% identity with *A. thaliana* HPR isoform 3 (AtHPR3) (33) and isoform 1 (AtHPR1) (34), respectively. Vv2KGR was also found to be highly homologous with *Coleus blumei* HPR (CbHPR, ∼78%) (35) and *Homo sapiens* HPR (GRHPR, ∼38%) (36). In addition to yiaE from *E. coli*, 2KDH proteins able to catalyze the reversible reduction of 2KLG to IA have recently been identified from *K. vulgare* bacteria (Kv2DH) (30) and fungus *A. niger* (gluC) (37). Vv2KGR has 41 and 33% amino acid identity (Fig. S1) with Kv2DH and gluC, respectively, which lends further support to the possibility that Vv2KGR may be able to catalyze 2KLG to IA.

### In vitro studies of enzymatic activity

**Figure 2.**
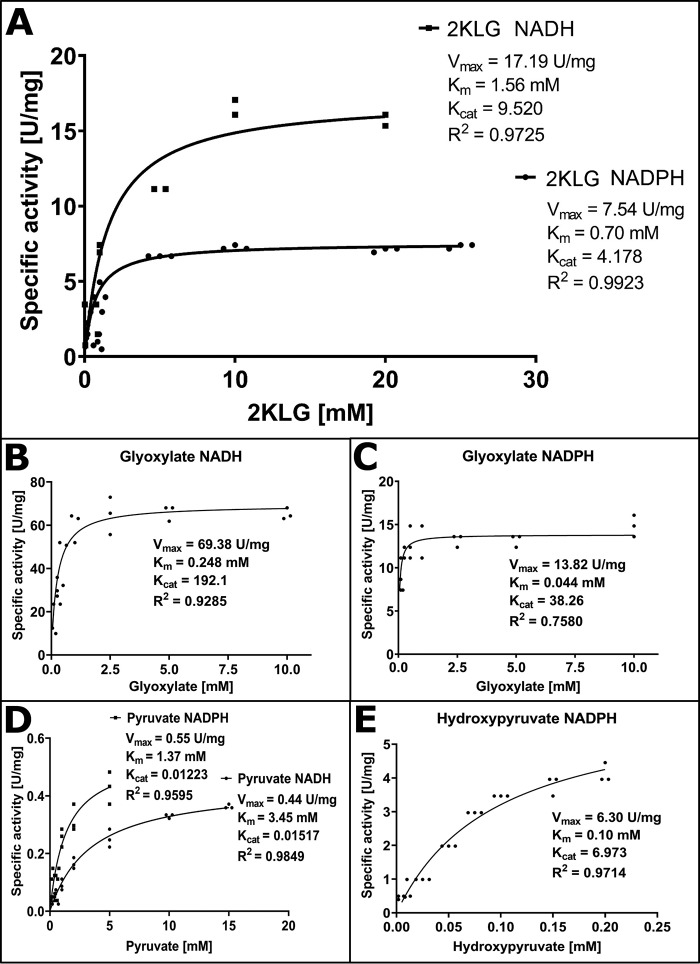
**Enzyme substrate specificity of Vv2KGR.** *A*, 2KLG with NADH and NADPH. All enzyme assay was performed in 100 mm HEPES, pH 7.5, with 1 mm NADH or 1 mm NADPH at 37 °C. *B*, glyoxylate with NADH. *C*, glyoxylate with NADPH. *D*, pyruvate with NADH and NADPH. *E*, hydroxypyruvate with NADPH (no activity could be detected with NADH). Each unit of enzyme activity is defined as the amount of the enzyme that catalyzes the conversion of 1 μmol of substrate/min.

Vv2KGR protein was expressed in an *E. coli* expression system and purified. The molecular mass of Vv2KGR was found to be around 33 kDa (Fig. S2), consistent with the computational prediction. To investigate whether Vv2KGR can catalyze the interconversion of 2KLG and IA, the speculated metabolic reaction in the degradation pathway of Asc to TA, 2KLG was tested as the substrate. A range of buffers and pH conditions were screened to determine the optimal enzymatic condition. Vv2KGR exhibited the highest activity at pH 7.5 in 100 mm HEPES buffer at 37 °C (Fig. S3). Vv2KGR was able to reduce 2KLG efficiently using both NADPH and NADH (Fig. 2*A*). The *K_m_* values for 2KLG with NADPH and NADH were determined as 0.70 and 1.561 mm, respectively, a clear preference for NADPH as the coenzyme. When NADPH was used as the coenzyme, *K*_cat_ of Vv2KGR on 2KLG was 4.18 s^−1^. As far as we are aware, this is the first report that a 2KDH protein with 2KLG reduction capacity is present in plants. The enzymatic profile of Vv2KGR is similar to *E. coli* yiaE, which also displays optimal activity at pH 7.5 and prefers NADPH over NADH (28). However, Vv2KGR and yiaE differ significantly from *A. niger* GluC, which is NADH-dependent and has no activity with NADPH (37). Furthermore, the *K_m_* and *K*_cat_ of GluC for 2KLG are 30 mm and 9.52 s^−1^, respectively, indicating a very divergent enzymatic profile for this fungal 2KDH.

In addition to 2KLG, the enzyme activities of Vv2KGR with glyoxylate (Fig. 2, *B* and *C*), pyruvate (Fig. 2*D*), and hydroxypyruvate (Fig. 2*E*) were also determined. Vv2KGR exhibited the highest activity at pH 7.5 in 100 mm HEPES buffer at 37 °C, similar to that reported for CbHPR (pH 7.0) (35) and GRHPR (pH 7.5) (36), but significantly different from the peroxisomal spinach HPR1 (pH 5.1 and pH 6.2 with NADPH and NADH, respectively) (38) and the cytosolic spinach HPR2 (pH 5.5–6.5) (39). Both NADH and NADPH could be used efficiently by Vv2KGR as the coenzyme for the reduction of glyoxylate, pyruvate, and 2KLG. Vv2KGR showed very little activity for hydroxypyruvate with NADH (substrate concentration tested: 0.05–25.0 mm). With NADPH, Vv2KGR displayed significantly lower *K_m_* values for all substrates tested (Fig. 2), indicating a clear preference for NADPH as the coenzyme. Both NADH-preferring and NADPH-preferring HPRs have been reported in plants, the latter for *C. blumei* HPR (35), *A. thaliana* HPR2 (33), spinach HPR2 (39), barley HPR-2 (40), and maize HPR (41). In addition, human GRHPR has also been shown to also prefer NADPH (36). Despite their NADPH preference, *A. thaliana* HPR2, barley HPR-2, and maize NADPH-preferring HPR still retained significant NADH-dependent hydroxypyruvate reduction activity. In contrast, we found no NADH-dependent hydroxypyruvate reduction activity for Vv2KGR, suggesting a distinct species-specific enzymatic property. A range of species-specific biological functions have been reported for plant HPRs. For example, HPR2 in *A. thaliania* (∼75% identity with Vv2KGR) has been shown to be involved in the photorespiration (32). In addition, CtHPR in *C. blumei* (78% identity with Vv2KGR) is able to catalyze the NADPH-dependent reduction of hydroxyphenylpyruvate, which participated in the biosynthesis of rosmarinic acid (35). 2KDH proteins, including plant HPRs, are known for their ability to catalyze the reduction of a series of 2-oxyacids to the d-isomers of the respective 2-hydroxyacids. The broad-substrate activity of Vv2KGR suggests that it may be involved in multiple biological pathways. The observation that Vv2KGR is able to reduce 2KLG efficiently using NADPH as the preferred coenzyme supports its biological role in TA biosynthesis from Asc.

### GC-MS confirmation of l-idonate formation by Vv2KGR

**Figure 3.**
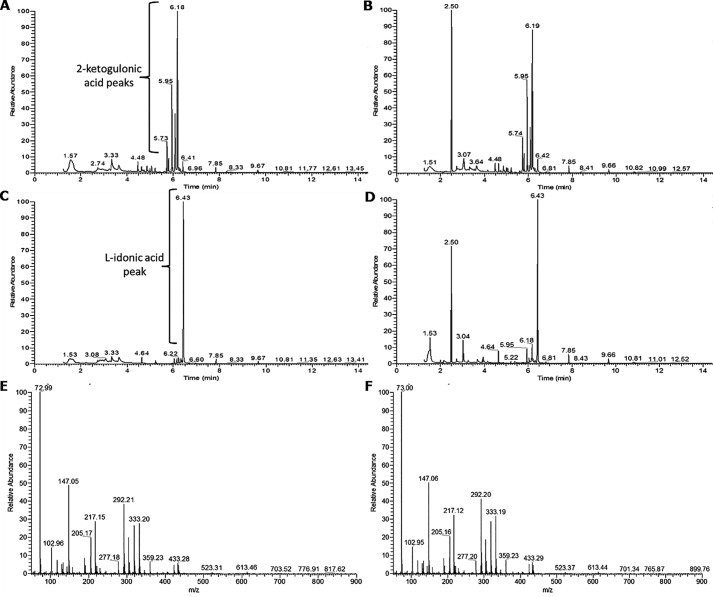
**Identification of Vv2KGR enzymatic assay products.** *A–D*, chromatograms for authentic 2-ketogulonic acid standard (*A*), enzyme assay product with denatured recombinant enzyme (*B*), authentic l-idonic acid standard (*C*), and enzyme assay product with intact recombinant enzyme (*D*). The main peak for l-idonic acid was detected at 6.43 min, and the *m*/*z* values are given for authentic l-idonic acid standard (*E*) and enzyme assay product (*F*).

GC-MS was used to identify the product of the Vv2KGR reaction with 2KLG as a substrate. The authentic 2KLG standard chromatogram had peaks at 5.73 min (predicted as 2-keto-d-gluconic acid, 36.29%, according to the NIST database), 5.95 min (2-keto-d-gluconic acid, 37.72%), 6.08 min (1H-indole, 40.45%; 2-keto-d-gluconic acid, 11.68%), and 6.18 min (1H-indole, 53.79%) (Fig. 3*A*). Reaction products from assays with denatured enzyme also showed these 2KLG peaks as well as an extra peak at 2.50 min that could be a breakdown product of Vv2KGR or something else present in the purified enzyme sample (Fig. 3*B*). The authentic l-idonate standard chromatogram had one major peak at 6.43 min (Fig. 3*C*). Reaction products from assays with intact enzyme also demonstrated a peak at 6.43 min, as well as some residual 2KLG peaks at 5.95 and 6.18 min, and the peak at 2.50 min observed previously with denatured enzyme reaction products (Fig. 3*D*). The *m*/*z* profile at 6.43 min was the same as that observed for the authentic l-idonate standard (Fig. 3, *E* and *F*). The same chromatogram and *m*/*z* profiles were obtained whether NADH or NADPH was used. Together, this demonstrates that recombinant Vv2KGR can convert 2-keto-l-gulonate to l-idonate in the presence of either NADH or NADPH and may therefore participate in the TA synthesis pathway of Asc degradation in *V. vinifera*.

### Gene transcriptional analyses

**Figure 4.**
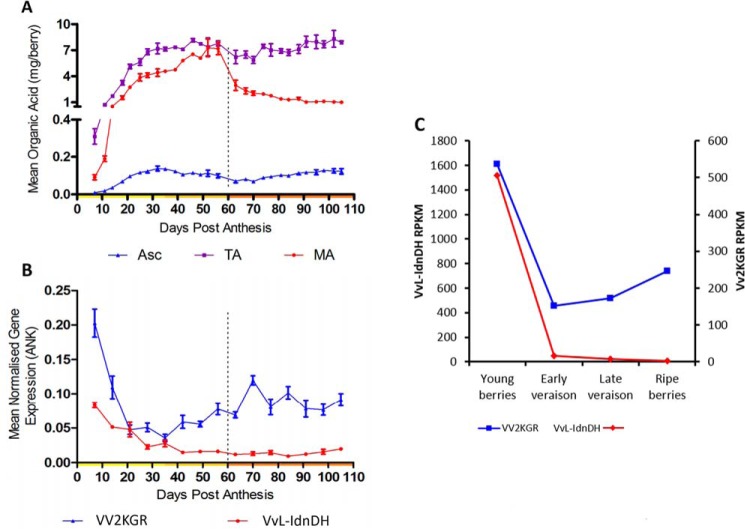
**TA accumulation and transcriptional analyses of *Vv2KGR* and *VvL-IdnDH* in developing grape berries.** *A*, TA (*purple*) content in developing *V. vinifera* cv. Shiraz berries, compared with malic acid (*MA*; red) and Asc (*blue*) (adapted from the data published by Melino *et al.* (1)). *B*, qRT-PCR analyses on *Vv2KGR* (*blue*) and *VvL-IdnDH* (*red*) against reference gene ankyrin (*ANK*). *C*, transcriptional profiles of *Vv2KGR* (*blue*) and *VvL-IdnDH* (*red*) based on RNA-Seq data by Sweetman *et al.* (6). *RPKM*, reads per kilobase per million mapped reads. *Error bars*, S.D.

TA is known to be rapidly synthesized in the early stage of grape berry development and remains stable throughout the ripening process. A developmental sample series of *V. vinifera* cv. Shiraz berries was used for measurement of organic acid contents alongside qRT-PCR analyses on *Vv2KGR* and *VvL-IdnDH*. TA content increased dramatically from 6 days post-anthesis (DPA) to 30 DPA and then plateaued before véraison (Fig. 4*A*). Transcript levels of both *Vv2KGR* and *VvL-IdnDH* were high in the early stage of berry development (Fig. 4*B*). *Vv2KGR* expression subsequently dropped to the lowest level at ∼35 DPA and then increased gradually toward maturation. In comparison, *VvL-IdnDH* transcription continued to decrease after 35 DPA and maintained a very low level. Taken together, the transcription pattern of Vv2KGR is consistent with the rapid increase of TA biosynthesis in the early stage of grape berry development. A similar pattern was seen for transcriptional profiles of *Vv2KGR* and *VvL-IdnDH* as extracted from a previously reported RNA-Seq data set in developing grape berry (Fig. 4*C*).

### X-ray crystal structure determination of Vv2KGR

To gain more insight into the biological function of Vv2KGR, the three-dimensional structure of Vv2KGR was determined by X-ray crystal structure analysis. The ligand-free Vv2KGR was crystallized in the primitive monoclinic space group P2_1_. The structure was solved to a resolution of 1.58 Å by the molecular replacement method. Data processing and refinement statistics are summarized in Table 1. Twinning analyses showed that the apo-Vv2KGR crystal was pseudo-merohedrally twinned. As such, a twinning refinement (twin operator: *h*, -*k*, -*l*) was applied during refinement, which allowed the structure to be refined to completion. Reduced model bias electron density quality can be viewed in the composite omit map (Fig. S4). Despite exhaustive co-crystallization and soaking attempts, no sufficient electron density could be observed for NADPH and 2KLG in the electron density map (Fig. S4).

**Table 1:** **Summary of X-ray diffraction data and refined model statistics for grapevine Vv2KGR crystal structure** Values in parentheses correspond to the last shell.

Parameters	Values
**Data collection and processing**	
Wavelength (Å)	0.9537
Space group	P2_1_
Unit-cell parameters (Å)	*a* = 73.05, *b* = 85.72, *c* = 112.90
	α = 90.00, β = 89.91, γ = 90.00
No. of observations	661,190 (27393)
Unique reflections	188,965 (9119)
*R*_merge_	0.091 (0.281)*^a^*
*R*_pim_	0.087 (0.262)
Completeness (%)	99.50 (97.5)
CC(1/2)	0.991 (0.919)
*I*/σ(*I*)	5.60 (1.5)
Multiplicity	3.50 (3.0)
**Refinement**	
Resolution range (Å)	1.58–27.70
*R*_work_ (%)	22.99*^b^*
*R*_free_ (%)	25.13*^c^*
Refined residues	1248 (312 × 4)
Water molecules	1077
Mean *B* factors (overall Å^2^)	20.99
RMSD bond angles (Å)	0.549
RMSD bond distances (Å)	0.002
**Protein geometry**	
Poor rotamers (%)	0
Ramachandran outliers (%)	0.16
Ramachandran favored (%)	94.84

*^a^R*_merge_ = Σ |*I* − < I >|/Σ*I*.

*^b^ R*_work_ = Σ|*F_o_* − *F_c_*|/Σ|*F_o_*| for all data excluding data used to calculate *R*_free_.

*^c^ R*_free_ = Σ|*F_o_* − *F_c_*|/Σ|*F_o_*|, for all data.

### Overall structure of Vv2KGR

**Figure 5.**
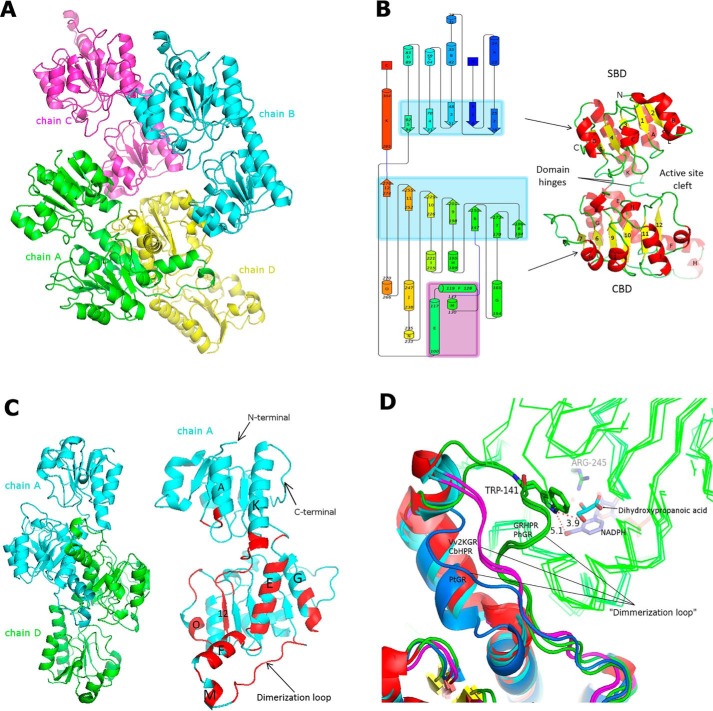
**Overall structure of Vv2KGR.** *A*, spatial arrangement of the four monomers of Vv2KGR in the asymmetric unit. *B*, *schematic graph* of the global folding of Vv2KGR monomer. β-Strand and α-helix clusters are *shaded* in *light blue* and *purple*, respectively. Residue numbers of each secondary structural element are labeled sequentially. *C*, dimer interface of Vv2KGR. The residues involved in the dimer interactions are annotated in *red*, with helix index *labeled. D*, structural variation of the dimerization loops for Vv2KGR and CbHPR (*pink*), GRHPR and PhGR (*green*), and PtGR (*marine blue*). Monomer 1 of each dimer is shown as *ribbon lines* (*green*). Dihydroxypropanoic acid (*cyan*) and NADPH (*purple*) in GRDPR are displayed as *sticks*. The potential interaction of Trp-141 (numbering according to GRHPR) in the dimerization loop of GRHPR and PhGR with the substrate (monomer 1) are displayed with distance (Å) *labeled*.

The determined Vv2KGR structure has four subunits (chain A–D) in the asymmetric unit (Fig. 5*A*), which share high structural similarity (Table S1). Vv2KGR monomer exhibits typical characteristics of the 2KDH family (Fig. 5*B*). Each subunit is composed of 15 α-helices and 12 β-sheets, which are arranged in a larger coenzyme-binding domain (CBD) and a smaller substrate-binding domain (SBD) (Fig. 5*B*). The two domains are joined by two loop hinges, a common observation in other 2KDH protein structures. Previous studies on the human GRHPR structure indicated that the flexibility of these two loop hinges could affect the enzyme catalysis process (36). Subunits of Vv2KGR demonstrate no significant angular changes between domains (∼2° between chain A and D), which were calculated by the DynDom program (42), suggesting a stable catalytic site. Vv2KGR has the highest structural similarity (all-atom RMSD 0.45 Å) with CbHPR (PDB entry 3BA1) and a relatively higher level of deviation with its animal and prokaryotic homologs (Table S1). In addition to HPR proteins, Vv2KGR structure also shares high homology with other 2KDH proteins, including d-lactate dehydrogenase, d-glycerate dehydrogenase, phosphoglycerate dehydrogenase, and transcription co-repression Ctbp dehydrogenase, with core RMSD values ranging from 1.17 to 4.40 Å (Table S1).

Both the CBD and SBD domains are marked by a typical α/β/α pattern (Fig. 5*B*), similar to that observed for 2KDH proteins. The CBD domain is composed of seven parallel strand sheets at the core, flanked by five and four α-helices, respectively, on each side, forming an NAD(P)H-binding Rossmann fold (Fig. 5*B*). This feature is also strictly conserved in the CbHPR (PDB code 3BA1) (43). Compared with the apo form CbHPR (PDB code 3BA1), Vv2KGR lacks an additional short α-helix (residues 207–209 in the apo-CbHPR structure), which is located adjacent to the substrate binding pocket (Fig. S5). This helix is also not observed in the binary CbHPR structure (PDB code 3BAZ, residues 207–209) (Fig. S5). A corresponding short α-helix was found in Kv2DH (PDB code 4LSW) and Ph2GR (PDB code 2DBZ) but not in human GRHPR (PDB code 2GCG) and *P. thermophile* Pt2GR (PDB code 2DBQ). The structural variation at this region may affect the substrate binding due to its proximity to the catalytic site. The SBD domain of Vv2KGR exhibited a 2-α-helix/5-β-strand/4-α-helix motif, forming a flavodoxin-like fold (Fig. 5*B*). This characteristic is strictly conserved in the CbHPR and Kv2DH structures. The short α-helix L (residues 29–31 in Vv2KGR, Fig. 5*B*) is not observed in human GRHPR, Ph2GR, and Pt2GR and may represent a unique structural characteristic for plant HPR proteins.

### Oligomeric interface

In the asymmetric unit, chain A forms extensive interactions with chain D, whereas chain B and chain C are positioned more closely with their respective crystallographic symmetry mates (Fig. 5*A*). This observation indicated that Vv2KGR may function as a biological dimer. Similar observations have been made for CbHPR, which functions as a dimer and shares high similarity with Vv2KGR (35, 43). Molecule contacts between subunits A and D of Vv2KGR resembled those observed in other 2KDH members. The interaction mainly involved residues from the CBD domains, including six helixes (E, F, G, K, M, and O), one β-strand (12), and several loops (Fig. 5*C*). One of these loops (residues 131–146 in Vv2KGR), inserted between helix M and sheet 6, has been defined as the dimerization loop (Fig. 5*C*). Sequence alignment showed that GRHPR has additional residues inserted at this position (Fig. S1). Structural comparison showed that the archaeal PhGR resembles human GRHPR and also demonstrates a longer dimerization loop positioned close to the active site, whereas PtGR has a relatively shorter dimerization loop than Vv2KGR and CbHPR (Fig. 5*D*). Moreover, the short helix M is missing in PtGR. Thus, it seems that the mammalian HPRs have a closer relationship with their archaeal counterparts, whereas the plant HPRs, represented by Vv2KGR and CbHPR, are also distinct from their bacterial homologs. These variations at the dimerization loop represent a notable difference among HPRs from different organisms and may have contributed to their distinct enzymatic profiles.

In addition to plant HPRs, other members of the 2KDH family, such as d-glycerate dehydrogenase (44), phosphoglycerate dehydrogenase (45), d-lactate dehydrogenase (46), transcription corepressor CtBP dehydrogenase (47), NAD(H)-dependent formate dehydrogenase (PDB code 3N7U), d-hydroxyisocaproate dehydrogenase (48), and d-mandelate dehydrogenase (PDB code 2W2K), from different organisms also form biological dimers. The exceptions are the phosphoglycerate dehydrogenases from *E. coli* and *Mycobacterium tuberculosis*, which form tetramers with distinct topologies (49, 50). The oligomeric interface of Vv2KGR was analyzed using the PDBePISA program (51). For the four subunits A, B, C, and D in the asymmetric unit, the buried solvent-accessible surface area (SASA) was calculated to be 2443 and 2428 Å^2^ for A-D and B-C interfaces, respectively, whereas the SASA for A-B, A-C, D-B, and D-C ranges from 235 to 458 Å^2^. A SASA cutoff of ∼850 Å^2^ has been used to discriminate true biological dimerization from nonspecific crystal contact (52). The oligomeric interface area calculation supports Vv2KGR as a biological dimer. In addition, oligomeric assembly analysis by PDEePISA identified stable quaternary structure for A-D and B-C only (PDB code 6PEX), providing clear evidence that Vv2KGR is a biological dimer. A total of 39 hydrogen bonds and eight salt bridges are formed at the interface (PDB code 6PEX), constituting the main force for the dimer interaction. This is relatively higher than the 28 hydrogen bonds and six salt bridges observed for the CbHPR dimer (PDB code 3BA1). A total of 23 residues are involved in hydrogen bonding interaction contributing to the dimer formation, the majority of which are conserved in CbHPR, except for residues 102, 124, 143, 165, 269, and 271 (Fig. S1). No disulfide bonds or covalent bonds were found between the two dimer subunits of Vv2KGR.

### Co-enzyme binding

**Figure 6.**
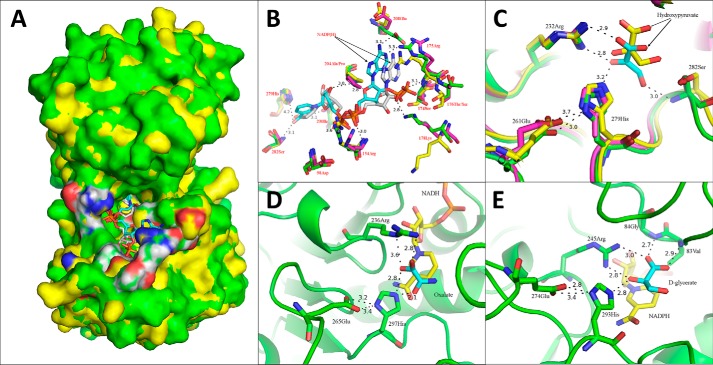
**Co-enzyme docking analyses of Vv2KGR and the two substrate-binding modes of 2KDHs.** *A*, overall positioning of the docked coenzyme NADPH (*cyan*) in Vv2KGR (*green*) in superimposition with CbHPR (*yellow*) complexed with NADPH (gray). The active-site residues are *highlighted* in *white* (carbon), *blue* (nitrogen), and *red* (oxygen). *B*, identification of the critical residues responsible for co-enzyme binding in Vv2KGR (*green*) and alignment with CbHPR (3BAZ (*yellow*) and 3BA1 (*magenta*)). The NADPHs for Vv2KGR and 3BAZ are *colored cyan* and *gray*, respectively. The potential hydrogen bond interactions with NADPH in Vv2KGR are indicated by *black dashed lines* with distance *labeled. C*, identification of the substrate-binding sites of Vv2KGR (*green*) in superimposition with CbHPR (*magenta*) and GRHPR (*yellow*). *D* and *E*, substrate-binding mode A (PDB code 2DLD; *L. helvveticus* d-lactate dehydrogenase bound with NADH (*yellow*) and oxalate (*cyan*)) and Mode B (PDB code 2GCG; GRHPR bound with NADPH (*yellow*) and d-glycerate (*cyan*)), respectively. The potential hydrogen-bonding interactions are indicated by *black dashed lines*.

To investigate the co-enzyme binding mechanism, NADPH was docked into the apo form Vv2KGR structure using the Molsoft Monte Carlo method (53). The obtained conformations with the lowest free energy were analyzed (Fig. 6, *A* and *B*).

The modeled Vv2KGR-NADPH complex was compared with the binary structure of CbHPR. A total of 10 potential hydrogen bonds were predicted between the modeled NADPH and Vv2KGR (Fig. 6*B*), which involves interactions with the main chains and side chains of residues (Arg-154, Arg-175, Lys-178, Ala-204, Glu-208, Ile-230, and Ser-282). This is significantly less than the 16 hydrogen bonds reported for NADPH in the CbHPR structure, in which additional residues (Asp-98, Ser-174, Ser-176, and His-279) also form hydrogen bonds with NADPH (43). Structural superimposition showed that most of the co-enzyme binding residues in CbHPR mentioned above were strictly conserved in Vv2KGR, except Ser-176 and Ala-204, which were replaced by Thr-176 and Pro-204, respectively (Fig. 6*B*). Sequence alignment showed that these NADPH-binding residues were highly conserved among plant HPR proteins and were also largely conserved in other 2KDH member proteins (Fig. S1). Significant side chain movements were observed for residues Arg-154, Ser-174, Arg-175, Ser-176, and Lys-178 upon coenzyme binding (Fig. 6*B*). Specifically, the side chains of Lys-178 moved dramatically away from the amide ring of NADP in the co-crystal of CbHPR, whereas the side chains of Arg-154, Ser-174, Arg-175, and Ser-176 were attracted to NADPH to form hydrogen bonds (Fig. 6*B*). Vv2KGR resembles the apo-form CbHPR at Arg-175, Ser-176, and Lys-178 but is more similar to the co-crystal of CbHPR at Arg-154. Vv2KGR displayed unique positions for Ser-174 and Glu-208 compared with the two forms of CbHPR, which may have a species-specific effect on the enzyme activity.

Most proteins from the 2KDH family employ NADH as the co-enzyme and have no activity with NADPH, such as the bacterial d-glycerate dehydrogenase (44), d-lactate dehydrogenase (46), formate dehydrogenase (54), phosphoglycerate dehydrogenase (55), and d-mandelate dehydrogenase (56). The enzyme assay in our study showed that Vv2KGR could utilize both NADH and NADPH as cofactor but displayed a clear preference for NADPH (Fig. 2). Similar results have been reported for CbHPR (35), human GRHPR (57), and Kv2DH (30), which also demonstrated substantially higher affinity for NADPH than NADH. Bernard *et al.* (58) identified the critical amino acid responsible for the absence of NADPH activity to be the Asp in a 2KDH protein from *Lactobacillus delbrueckii* subsp. Bulgaricus, which corresponds to Ser-174 in Vv2KGR (Fig. 6*B*). The negatively charged carboxyl group of the Asp side chain, positioned at the co-enzyme binding site, was assumed to prevent the binding of NADPH due to its similarly negatively charged phosphate group (58). The Asp is commonly conserved in the NAD(H)-dependent 2KDHs (Fig. S1). In our modeled Vv2KGR-NADPH complex, the oxygen atom of the carboxyl group in Ser-174 was positioned close (3.1 Å) to the phosphate group of NADPH with hydrogen bonding potential (Fig. 6*B*), which may facilitate the preferential binding of NADPH over NADH. Structural superimposition of Vv2KGR with CbHPR, human GRHPR, and Kv2DH revealed a highly conserved Ser residue at this site (Fig. 6*B*). CbHPR (PDB code 3BAZ) and human GRHPR (PDB code 2GCG) have over 20-fold higher affinity for NADPH than with NADH. The Ser-174 in the NADP(H) co-crystals of both CbHPR and GRHPR also form hydrogen bonds to the phosphate group of the bound NADP(H) molecule, which may contribute to a higher NADP(H)-binding affinity for these proteins. The coenzyme-binding affinity for NADP(H)-dependent 2KDH proteins displays significant variations. For example, CbHPR has much higher affinity (*K_m_* = 0.02 mm) (35) for NADP(H) than does GRHPR (*K_m_* = 0.11 mm) (57), whereas the *K_m_* value of Kv2DH for NADH (0.11 mm) was just over twice that for NADPH (0.05 mm) (30). Taken together, these observations indicated that, whereas the Asp-174-to-Ser substitution favors the binding of NADP(H), the absolute binding affinities for NAD(H) and NADP(H) of 2KDHs are also organism-dependent.

### Catalytic mechanism and substrate-binding modes of Vv2KGR

Three electrostatically charged amino acids (Arg-232, Glu-261, and His-279 in Vv2KGR) have been identified for substrate binding in previous studies on 2KDH structures, which correspond to Arg-232–Glu-261–His-279 in CbHPR, Arg-269–Glu-274–His-293 in GRHPR, and Arg-234–Glu-263–His-281 in Kv2DH (Fig. 6*C*). These three active-site residues are commonly conserved in most other 2KDH members, including d-glycerate dehydrogenase, d-lactate dehydrogenase, phosphoglycerate dehydrogenase, phosphite dehydrogenase, transcription co-repression Ctbp dehydrogenase, d-mandelate dehydrogenase, and d-2-hydroxyisocaproate dehydrogenase (Fig. S1). As shown in Fig. 6*C*, the OE1 and OE2 atoms of Glu-261 in Vv2KGR are hydrogen-bonded (3.7 and 3.0 Å) with the ND1 atom of His-279. This hydrogen bonding interaction was suggested to establish a charge relay system (36, 43). It helps to protonate the imidazole ring of His-279, which acts as the acid/base catalyst during the catalytic process and attracts electron flow from the C2 carbonyl group of the substrate. 2KDH proteins have been known for their broad substrate specificity and are able to catalyze the reduction of a series of 2-oxyacids to the d-isomers of the respective 2-hydroxyacids. The side chain of Arg-232 in Vv2KGR adopts a similar position with the catalytic Arg residues in CbHPR and human GRHPR (Fig. 6*C*). Potential hydrogen-bonding interactions between the side chain of Arg-232 and the carboxyl oxygen atom of the bound substrate can also be observed in Vv2KGR, which may help to orient the 2-keto group for electron attack, leading to the production of 2-hydroxyl acid.

Two distinct substrate binding modes (A and B) have been reported for 2KDHs in previous studies. The first binding mode A was deduced from the binary structure of the *Lactobacillus helvveticus* d-lactate dehydrogenase (PDB code 2DLD). As shown in Fig. 6*D*, the side chain of Arg-236 in *L. helvveticus* d-lactate dehydrogenase forms two hydrogen bonds with the two oxygen atoms of the carboxyl of oxamic acid. An electrostatic interaction is also believed to exist between the positively charged Arg-236 and the electro-negative carboxyl group. The oxamic acid substrate is further stabilized by the hydrogen bond between the oxygen atom of the C2 and the ND2 atom from the His imidazole ring. The nicotine amide ring of the coenzyme is positioned under the panel formed by the substrate and its coordinating residues, which makes the hydride transfer from NADH to the C2 keto group of the substrate possible (Fig. 6*D*). Binding mode A has been observed in the acetic acid-bound human CtBP structure (PBD code 1MX3; 25% identity with Vv2KGR) and the human d-3-phosphoglycerate dehydrogenase structure (PDB code 2G76; NADH and malate bound; 32% identity with Vv2KGR).

In addition to binding mode A, an alternative substrate binding mode B has also been observed in human GRHPR structure (Fig. 6*E*). In mode B, although the highly conserved catalytic Arg-245 still coordinates the substrate (d-glyoxylate) with two hydrogen bonds, only one oxygen atom of the carboxyl group is involved. The other hydrogen-bonding oxygen atom belongs to the C2 carbonyl group (Fig. 6*E*). For the GRHPR structures, the C2 oxygen atom of d-glycerate also forms a hydrogen bond with the ND2 of the imidazole ring of the catalytic His-293, facilitating the keto reduction at the C2 position. Binding mode B is supported by the modeled tertiary structure of a *Lactobacillus pentosus* d-lactate dehydrogenase (PDB code 1GDH) (59) and a *Lactobacillus bulgaricus* d-lactate dehydrogenase (PDB code 1J49) (60). The spatial arrangement of the predicted pyruvate for 1GDH and 1J49 resembles the spatial coordination observed for d-glyoxylate in human GRHPR, with two oxygen atoms from the carboxyl group and C2 carbonyl group, respectively, oriented to the side chain of Arg by hydrogen bonding interactions (59, 60). Later studies on the *Aquifex aeolicus* d-lactate dehydrogenase (PDB code 3KB6; NAD and d-lactic acid bound; 29% identity with Vv2KGR) lend direct support for binding mode B.

Recently, the simultaneous occurrence of binding modes A and B has been reported in the human CtBP structure (PDB code 4U6S; NAD^+^ and phenylpyruvate bound; 25% identity with Vv2KGR) (61). In binding mode A, the two carboxylate oxygen atoms of phenylpyruvate are oriented to the side chain of the catalytic Arg-266 within hydrogen-bonding distance, similar to *L. helvveticus* d-lactate dehydrogenase (PDB code 2DLD). In binding mode B, the phenylpyruvate carbonyl is coordinated by two hydrogen bonds with the side chains of Arg-266 and His-315, respectively. Only one oxygen atom from the C1 carboxyl group has hydrogen-bonding potential with Arg-266. This resembles the spatial arrangement of d-glyoxylate in human GRHPR. The presence of two conformations at the same time provides direct evidence that both substrate binding modes are possible for 2KDH proteins in the native structure. Depending on the specific active site environment and also the substrate molecule property, different proteins may adopt specific substrate binding modes to a variable degree. Recently, computational docking analysis CbHPR supported the possibility of two binding modes (43), demonstrating comparable predicted free energies for several validated native substrates. The structural information from the phenylpyruvate-bound human CtBP (two substrate-binding modes present simultaneously) suggests that only binding mode B would facilitate C2 keto reduction, leading to the production of the R-isoform product. In binding mode A, hydride transfer to the C2 carbonyl from the nicotinamide ring of the coenzyme becomes impossible. Similar conclusions have been made for the human GRHPR, CbHPR, *L. bulgaricus* d-lactate dehydrogenase (60), and *A. aeolicus* d-lactate dehydrogenase (46). This may represent the distinct substrate-binding features between 2KDH proteins and the l-specific oxidoreductases, such as l-lactate dehydrogenase. Vv2KGR demonstrates relatively strong homology with CbHPR, GRHPR, PhGR, and Kv2DH. Structural superimpositions of Vv2KGR with these proteins revealed highly conserved active site residues, suggesting that two substrate binding modes may be present in Vv2KGR, whereas only binding model B would be functional.

### Molecular docking and substrate specificity

To investigate the substrate binding mode of Vv2KGR and its substrate specificity, d-glyoxylate, pyruvate, 3-hydroxypyruvate (commonly accepted substrates for HPR proteins), 2KLG (the proposed Vv2KGR substrate in the TA synthesis pathway in grapevine), and l-ribulosonic acid (a five-carbon homolog of 2KLG) were docked into Vv2KGR.

**Figure 7.**
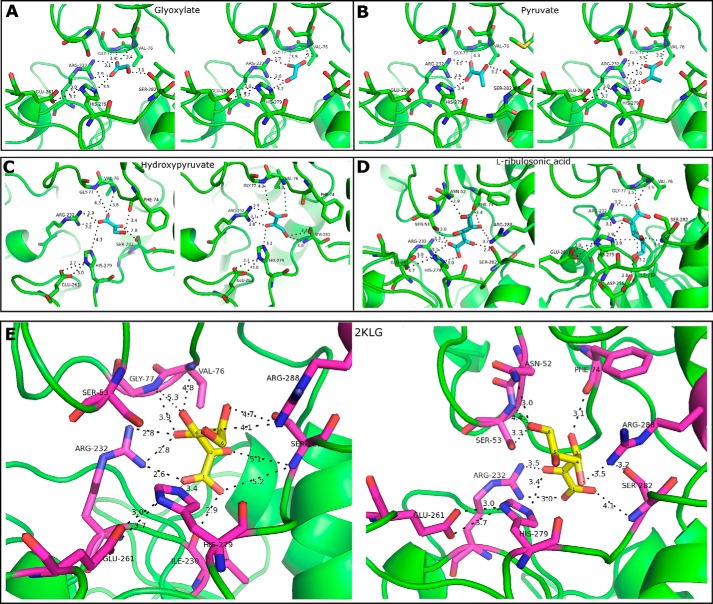
**Substrate-docking analyses for Vv2KGR.** Two binding modes are displayed for d-glycerate (*A*), pyruvate (*B*), hydroxypyruvate (*C*), l-ribulosonic acid (*D*), and 2KLG (*E*). For each *panel*, binding mode A and mode B are shown on the *left* and *right*, respectively. The identified critical amino acids for substrate binding are shown in *sticks*. The potential hydrogen-bonding interactions coordinating the substrates are indicated by *black dashed lines* with distance (Å) labeled. Amino acid numbering is according to Vv2KGR.

Overall, the degree of flexibility in substrate binding increased with the number of the carbon atoms in the ligand (Table 2). This also coincided with a decreasing predicted substrate free energy, which may be attributed to the binding interaction with the additional hydroxyl groups. Similar results have been reported for the substrate docking analyses for CbHPR (43). The top binding conformations recognized as mode A and B for each substrate are displayed in Fig. 7. Vv2KGR displays the lowest predicted free energy for 2KLG (Table 3). All of the five substrates studied demonstrate a lower predicted free energy for binding mode B, suggesting a binding preference for mode B over mode A. For all five substrates, the C2 ketone oxygen atom in binding mode A was positioned away from the nicotinamide ring of the His-279 side chain, which acts as the catalytic base (Fig. 7). This would hinder the reduction at the C2 position, suggesting that binding mode A may not be biologically functional for Vv2KGR. In addition to the above mentioned active triad Arg-232–Glu-261–His-279, residues Asn-52, Ser-53, Phe-74, Ser-282, and Arg-288 were also predicted to coordinate 2KLG in our docking results, indicating that these amino acid sites may also be involved in the catalytic process (Fig. 7).

**Table 2:** **Enzymatic activity of Vv2KGR with different substrates** Each enzymatic unit was defined as the amount of the enzyme that catalyzes the conversion of 1 μmol of substrate/min under the tested assay condition. −, no activity.

Substrate	Coenzyme	*K_m_*	*V*_max_	*K*_cat_
		*mm*	*units/mg*	*s*^−*1*^
d-Glyoxylate	NADH	0.248	69.380	192.10
	NADPH	0.044	13.820	38.26
Pyruvate	NADH	3.453	0.442	0.01517
	NADPH	1.371	0.548	0.01223
Hydroxypyruvate	NADH	−	−	−
	NADPH	0.096	6.295	6.973
2KLG	NADH	1.561	17.190	9.520
	NADPH	0.700	7.544	4.178

**Table 3:** **Summary of the docking results** Binding modes A and B are defined under “Results and Discussion.”

Substrate	Generated conformations	Binding mode	Predicted free energy	Binding mode frequency*^a^*	Predicted product stereochemistry
			*kcal/mol*		
d-Glyoxylate	11	B	−17.96	3/11	R
		A	−17.94	5/11	NP*^b^*
Pyruvate	9	B	−22.38	4/9	R
		A	−22.19	5/9	NP
3-Hydroxypyruvate	20	B	−27.01	12/20	R
		A	−26.56	7/20	NP
l-Ribulosonic acid	47	B	−35.60	4/47	R
		A	−33.29	5/47	NP
2KLG	54	B	−39.77	11/54	R
		A	−37.52	3/54	NP

*^a^* Binding mode frequency was calculated as the number of conformations for each binding mode divided by the total number of binding conformations obtained.

*^b^* NP, no protonation.

The docked 2KLG exhibited more hydrogen bond interactions than the other studied substrates (Fig. 7). A lower predicted free energy would indicate a higher binding affinity for 2KLG. Therefore, it is reasonable to speculate that Vv2KGR is able to catalyze the *in vivo* interconversion between 2KLG and l-idonic acid in the Asc degradation pathway. This resembles the case for CbHPR in the rosmarinic biosynthesis. CbHPR identified from *C. blumei* is involved in the reduction of 4-hydroxyphenylpyruvate in the rosmarinic biosynthesis pathway, although CbHPR displayed a substrate preference for hydroxypyruvate over 4-hydroxyphenylpyruvate (43). Substrate docking analyses revealed a lower predicted free energy for 4-hydroxyphenylpyruvate than hydroxypyruvate for CbHPR (43). A notable characteristic for 2KDH proteins is their broad substrate specificity, which suggests that these proteins may have multiple functions in different metabolic pathways across different organisms. For example, bacterial homologous enzymes such as *E. coli* yiaE (28) and *K. vulgare* 2DH (30) in the ketogluconate metabolic pathway have also been shown to be able to reduce 2KLG to l-idonic acid. Both of these two enzymes belong to the 2KDH family. The crystal structure of *K. vulgare* 2DH (PDB code 4LSW; 41% sequence similarity) has also been determined, which displayed highly conserved substrate-binding sites with Vv2KGR.

It was reported that human GRHPR has a much higher affinity for hydroxypyruvate over pyruvate or glyoxylate (57). The preference for hydroxypyruvate by human GRHPR might be due to potential hydrogen bond interactions between the hydroxymethyl in hydroxypyruvate and residues Ser-296, Trp-141 (from the dimer molecule), whereas the lower binding affinity for pyruvate and glyoxylate may be attributed to their steric clash with Leu-59 (36). Structural comparison of Vv2KGR with human GRHPR showed that only Ser-296 was conserved in Vv2KGR, whereas Leu-59 and Trp-141 were replaced by Ser-59 and Gly-141, respectively. In GRHPR, Trp-141 in the dimerization loop is positioned close to the substrate-binding pocket. Based on this observation, Booth *et al.* (36) suspected that the substrate specificity of GRHPR might be dependent on the dimerization process of the enzyme. Indeed, we observed that the corresponding dimerization loops of Vv2KGR and CbHPR are much shorter and positioned away from the substrate binding sites (Fig. 5*D*), which may lead to reduced binding affinity for the substrates.

### Conclusion

We have identified an aldo-keto reductase Vv2KGR with 2-keto-l-gulonic acid reductase activity from grapevine. Vv2KGR displays strong homology with *E. coli* yiaE, *K. vulgare* Kv2DH, and *A. niger* gluC, which are able to catalyze the conversion of 2KLG to IA in gluconic acid metabolism. Vv2KGR can reduce 2KLG to IA efficiently using NADPH as the preferred coenzyme. In addition, the transcription profile of Vv2KGR was consistent with TA accumulation in developing grape berries, supporting its potential role in TA biosynthesis from vitamin C in grapevine. We determined the crystal structure of Vv2KGR to a resolution of 1.58 Å. The catalytic sites of Vv2KGR are highly conserved compared with other protein structures in the 2KDH superfamily. The catalytic mechanism of Vv2KGR on 2KLG reduction was investigated by substrate docking, which revealed the favorable binding mode for 2KLG. This is the first report that a 2KDH capable of converting 2KLG to IA is present in plants.

## Experimental procedures

### Chemicals

Chemicals used for enzymatic tests and GC-MS were purchased from Sigma, BDH, Merck, and Gold Biotechnology. All samples were of analytical grade or higher. l-Idonate (sodium salt) was obtained previously from Kazumi Saito (Kyoto University, Kyoto, Japan). Buffers, enzyme substrates, and coenzyme stocks were prepared in deionized water.

### Identification of a putative 2-keto-l-gulonate reductase gene from grapevine

The amino acid sequence of the *E. coli* yiaE (UniProt number P37666) (28) was used as a query to tBLASTn (*E*-value = 0) against the NCBI Gene Index Project: Gene Indices: Grape EST database. The encoded amino acid sequences of the sequence hits were analyzed for the presence of d-isomer 2-hydroxyacid reductase and NAD(P)H-binding domain signatures using the Interpro online tool (http://www.ebi.ac.uk/interpro/).^4^

### RNA extraction, cDNA synthesis, and molecular cloning

RNA was extracted from pre-verasion *V. vinifera* cv. Shiraz berries using RNeasy Mini kit (Qiagen, Chadstone, Australia). cDNA was synthesized using SuperScript® III reverse transcriptase and oligo(dT)_20_ primer (Invitrogen). Gene-specific primers containing NdeI (forward, CTACATATGATGGCGATGATGAAGCGAGTTGCTGAG) and BamHI (reverse, CCAGGATCCTTACTGGTACTGGCTTGCTAGTTGGCC) restriction sites were designed to clone *Vv2KGR*. PCR was performed using the Phusion® High-Fidelity DNA polymerase (New England BioLabs). Digested PCR product was ligated into the pDRIVE (Qiagen, Germany) vector for sequencing confirmation. The putative *Vv2KGR* gene was then cloned into the pET14b (Novagen, Germany) vector and was transformed into BL21(DE3) pLysS-T1® (Sigma-Aldrich) for recombinant protein production.

### Recombinant protein expression and purification

*E. coli* strain harboring the pET14b-Vv2KGR construct was grown in LB culture (10 liters) containing 50 μg/ml ampicillin with shaking (200 rpm) at 37 °C until the *A*_600_ reached 0.6. Protein expression was initiated with the addition of 0.5 mm isopropyl 1-thio-β-d-galactopyranoside. Induction was carried out at 16 °C overnight. Cells were harvested by centrifugation and disrupted mechanically using a French press. The lysate was centrifuged at 4 °C, 40,000 × *g* for 30 min. The supernatant was loaded onto a HisTrap FF Crude 5 × 1-ml column (GE Healthcare) using an NGC^TM^ chromatography system (Bio-Rad). Eluted fractions containing the target protein were combined and loaded onto a Hitrap Q HP, 5-ml column (GE Healthcare) to further remove contaminant proteins. The fractions containing the target protein were dialyzed overnight at 4 °C. Part of the resulting enzyme solution was mixed with 10% glycerol and stored at −80 °C for later enzyme assays, whereas the rest was concentrated using an Amicon Ultra-15 centrifugal filter (Merck Millipore) for crystallization. The washing and elution buffers used for the His-tagged purification were 20 mm Tris (pH 8.0), 500 mm NaCl, 1 mm 2-mercaptoethanol, 10 mm imidazole and 20 mm Tris (pH 8.0), 500 mm NaCl, 1 mm 2-mercaptoethanol, 250 mm imidazole, respectively. The washing and elution buffers used for the Q anion-exchange column were 20 mm Tris (pH 8.0), 50 mm NaCl, 1 mm DTT and 20 mm Tris (pH 8.0), 750 mm NaCl, 1 mm DTT, respectively. The dialysis buffer contained 20 mm Tris (pH 8.0), 50 mm NaCl, and 1 mm DTT. The elution fractions containing the target protein were checked on a 10% SDS-polyacrylamide gel stained by Coomassie Brilliant Blue G-250.

### Enzyme kinetic assay

The activity of recombinant Vv2KGR was monitored at 340 nm, 37 °C in a 96-well flat-bottom UV plate (Costar) using a FLUOstar Omega spectrophotometer (BMG Labtech, Ortenberg, Germany). Each reaction contained 187 μl of buffer solution, 2 μl of coenzyme solution, 1 μl of enzyme sample, and 10 μl of substrate solution. All assays were carried out in triplicate except the test of 2KLG with NADPH, for which duplication was performed. A master reagent mix including purified enzyme, buffer, and coenzyme was dispensed into the 96-well plate. The optimal pH condition was determined using a range of buffers: 100 mm HEPES pH 6.5–7.0–7.5–8.0, 100 mm Tris-HCl pH 6.0–6.5–7.0–7.5–8.0–8.5–9.0, 100 mm and NaOH-glycine pH 8.0–8.5–9.0–9.5–10.0. Negative controls with no substrate were also included. The rate of absorbance changes was processed using MARS data analysis software (version 3.10 R6, BMG LABTECH) and was converted to the quantity of specific enzyme activity using an extinction coefficient of 6.22 mm^−1^ cm^−1^ for NADH and NADPH. The obtained initial reaction rates at different substrate concentrations were fitted into the Michaelis–Menten equation using GraphPad Prism software (version 6.0) to calculate the *K_m_*, *K*_cat_, and *V*_max_.

### Plant materials and qRT-PCR

The fruit samples of *V. vinifera* cv. Shiraz used in the present study were sampled by Melino *et al.* (1). qRT-PCRs were conducted on a fee basis by the South Australian Research and Development Institute (SARDI), using an ABI HT7900 Fast Real Time PCR system. Three biological samples were analyzed, each with three technical replicates. Results were analyzed using Sequence Detection System version 2.3 software (Applied Biosystems). Ankyrin was used as the reference genes using primers designed by Sweetman *et al.* (6). Gene-specific primers (forward, GACAAGATCGATTTGGTGAGG; reverse, CATCCGGAGTGTTCGTAACC) for *Vv2KGR* were designed in this study. Each sample reaction contained 0.9 μm (final) primer, 0.2 pmol (final) of probe, 50 ng (final) of cDNA, and 1× FastStart Universal Probe Master Mix to a final volume of 16 μl. The cycling conditions used were as follows: 95 °C for 10 min, 95 °C for 15 s, 57 °C for 1 min. Steps 2 and 3 were cycled 45 times. Quantification was achieved via a standard curve of known concentration samples (1, 10^−2^, 10^−4^, 10^−5^, 10^−6^, 10^−7^, 10^−8^, and 10^−9^ fmol). Normalization was performed using reference genes ankyrin and ubiquitin.

### GC-MS analyses

Purified Vv2KGR enzyme was used in an enzyme assay, which was followed until completion using a UV spectrophotometer at 340 nm under optimized conditions (based on kinetic analyses). Extra substrate and coenzyme were added numerous times, until the enzyme stopped functioning. Then the reaction products were derivatized and analyzed by GC-MS using methods described previously (62). The same conditions were used with denatured enzyme, and the product of this reaction was also tested.

### Protein crystallization

Crystals were grown in sitting drops by vapor diffusion in 96-well INTELLI-PLATE (Art Robbins Instruments) at 16 °C. The protein sample used for crystallization was concentrated to ∼10 mg/ml in the above-mentioned dialysis buffer. Each well consisted of a 1-μl/1-μl ratio of well solution to protein solution and the well solution reservoir (75 μl). The screening plates were set up using a Phoenix robot system (Art Robbins Instruments). In addition to the ligand-free sample, an enzyme solution containing 2 mm NADPH was also tested to obtain NADPH-Vv2KGR co-crystals. Crystals were obtained in a number of solutions in the Wizzard Classic 1 and 2 block (Rigaku) and PEG/ion screen (Hampton Research). The protein crystals used for diffraction were obtained in solution containing 1.26 m ammonium sulfate, 100 mm sodium acetate/acetic acid (pH 4.5), and 200 mm sodium chloride.

### Data collection, structure determination, and refinement

Crystals were transferred to methyl-2,4-pentanediol at ∼22.5% (w/v) and were flash-cooled to 100 K by submersion into liquid nitrogen. Data collection was performed at 100 K on the MX1 beamline at the Australian Synchrotron (Victoria, Australia) (63). The peak wavelength was at 0.9537 Å. Complete data sets were obtained with a rotation of ▵ϕ = 0.5°. Data were indexed, integrated, and scaled using the iMosflm and Scala programs within the CCP4 package. The space group was determined as P2_1_, and data were processed to a resolution of 1.58 Å. The phase problem was solved by molecular replacement method using the program Phaser MR (64). A single best solution was obtained, with a log-likelihood gain value of 20,809. A homology model of Vv2KGR was created using ICM Pro (Molsoft LLC, La Jolla, CA) with the protein structure of CbHPR (PDB code 3BAZ) as the search model. Data quality analysis using Phenix.xtriage revealed the presence of twinning in the crystal structure, with the twin law *h*, -*k*, -*l* and a twinning fraction of 0.35. The generated model was then refined using Phenix.refine and Coot until *R* factors reached convergence. Phenix was used to generate *R*_free_ flags and was used throughout refinement. As such, the twin law was used to assign consistent *R*_free_ flags. Additionally, Phenix refines the twin ratio throughout the refinement. Structural comparisons of Vv2KGR with other 2KDH proteins were performed using Coot and PyMOL (version 1.3r1; Schrodinger, LLC, New York).

### Substrate docking

Substrate docking was performed in ICM Pro (Molsoft LLC, La Jolla, CA) using the interactive docking module. Ligand molecules were downloaded either from the RCSB Protein Data Bank or from ChemSpider (http://www.chemspider.com/)^4^ and were then loaded into ICM Pro for electrostatic charge assignment (formal charges), pH setup (pH 7.0), and energy minimization. Each substrate conformation was analyzed and ranked according to its predicted free energy. Hydrogen-bonding interaction potentials with the active site residues were examined manually. All molecular visualizations were obtained using the PyMOL graphic tool. The deduced reaction mechanism of Vv2KGR on the reduction of 2KLG was drawn using the Marvin online tool.

## Author contributions

C. M. F., J. B. B., and K. S. supervised the project and provided valuable comments on manuscript development. Y. J. substantially wrote the manuscript. Y. J., C. A. B., C. S., and E. S. performed gene cloning, protein purification, and enzymatic tests. C. A. B., K. S., and C. J. were responsible for TA measurement and RT-PCR. R. D. H. and C. S. conducted GC-MS and RNA-Seq data mining. Y. J. and J. B. B. performed protein crystallization, model building, substrate docking, and data analyses.

## Supplementary Material

Supporting Information

## Footnotes

4 Please note that the JBC is not responsible for the long-term archiving and maintenance of this site or any other third party hosted site.

## Footnotes

3 The abbreviations used are:

TA: l-tartaric acid
Asc: ascorbic acid
DHA: dehydroascorbate
2KLG: 2-keto-l-gulonic acid
IA: l-idonic acid
2KDH: d-isomer–specific 2-hydroxyacid dehydrogenase
HPR: hydroxyl pyruvate reductase
DPA: days post-anthesis
CBD: coenzyme-binding domain
SBD: substrate-binding domain
RMSD: root mean square deviation
CtBP: C-terminal binding protein
SASA: solvent-accessible surface area
EST: expressed sequence tag
PDB: Protein Data Bank
qRT-PCR: quantitative RT-PCR.
