# Supplementary material for: An aldo-keto reductase with 2-keto-l-gulonate reductase activity functions in l-tartaric acid biosynthesis from vitamin C in Vitis vinifera
Source: J Biol Chem. 2019 Sep 4;294(44):15932–46. doi: 10.1074/jbc.RA119.010196 (PMC6827314; doi:10.1074/jbc.RA119.010196)
Supplement: Supporting Information [file supp_294_44_15932__index.html]

An aldo-keto reductase with 2-keto- L-gulonate reductase activity functions in L-tartaric acid biosynthesis from vitamin C in Vitis vinifera — Identification of a reductase for tartaric acid biosynthesis — An aldo-keto reductase with 2-keto-l-gulonate reductase activity functions in l-tartaric acid biosynthesis from vitamin C in Vitis vinifera — Identification of a reductase for tartaric acid biosynthesis — Supporting Information 

# An aldo-keto reductase with 2-keto-l-gulonate reductase activity functions in l-tartaric acid biosynthesis from vitamin C in *Vitis vinifera*

## Supporting Information

- Supporting Information (to be published online) - Supplementary Material
