# Supplementary material for: An aldo-keto reductase with 2-keto-l-gulonate reductase activity functions in l-tartaric acid biosynthesis from vitamin C in Vitis vinifera
Source: J Biol Chem. 2019 Sep 4;294(44):15932–46. doi: 10.1074/jbc.RA119.010196 (PMC6827314; doi:10.1074/jbc.RA119.010196)
Supplement: Supporting Information [file supp_RA119.010196_154310_1_supp_386788_px09ms.pdf]

Supplementary Materials

Title: An aldo keto reductase with 2-keto- L-gulonate reductase activity functions in L-tartaric acid biosynthesis from vitamin C in Vitis vinifera

Authors: Yong Jia, Crista A Burbidge, Crystal Sweetman, Emi Schutz, Kathy Soole, Colin Jenkins, Robert D. Hancock, John B. Bruning, Christopher M. Ford

Corresponding authors: **Christopher M Ford**: School of Agriculture, Food & Wine, University of Adelaide, Adelaide, Australia, 5064; christopher.ford@adelaide.edu.au; Tel. +61 08 8313 7386. **John B Bruning**: School of Biological Sciences, University of Adelaide, Adelaide, Australia, 5005; john.bruning@adelaide.edu.au; Tel. +61 08 8313 5218.

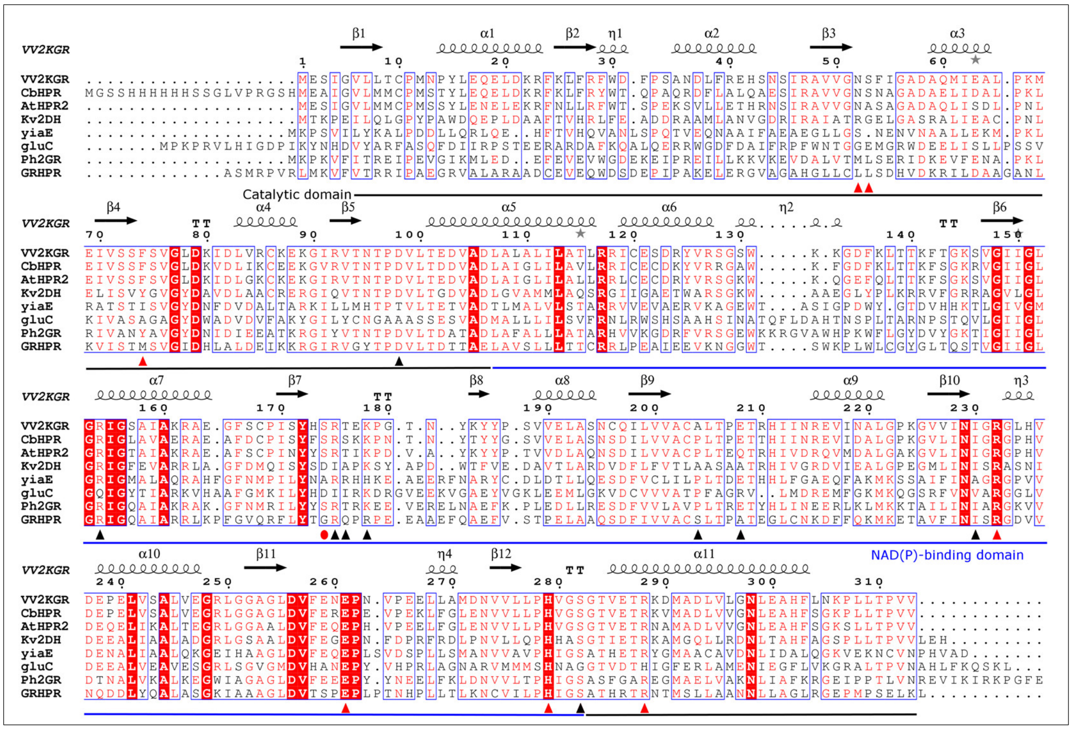

Figure S1. Amino acid sequence alignment of Vv2KGR with homologous 2KDHs. The amino acid sequences of Vv2KGR and other 2KDHs: *C. blumei* CbHPR, *A. thaliana* AtHPR2, *K. vulgare* Kv2DH, *E. coli* yiaE, *A. niger* gluC, *P. horikoshii* Ph2GR and human GRHPR were aligned. The catalytic sites identified for NADP-binding (black triangle), 2KLG binding (red triangle), the catalytic domain (blue underline) and NAD(P)H-binding domain (black underline) in Vv2KGR were indicated. The amino acid residue (174) for NADH/NADPH selection was indicated by solid red dot. The secondary structural elements α-helices (ᘐᘐ), β-strands (→) and strict β-turns (TT) were indicated on top based on Vv2KGR structure. Amino acid numbering is according to Vv2KGR.

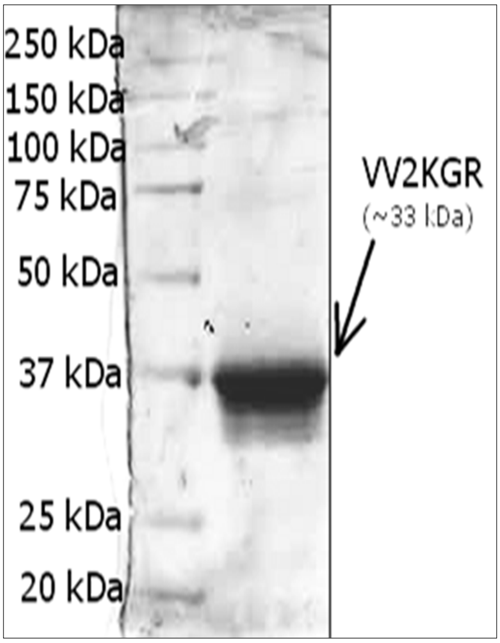

Figure S2. SDS-PAGE gel displaying the purified recombinant Vv2KGR. The estimated protein size for Vv2KGR is ~ 33 kDa.

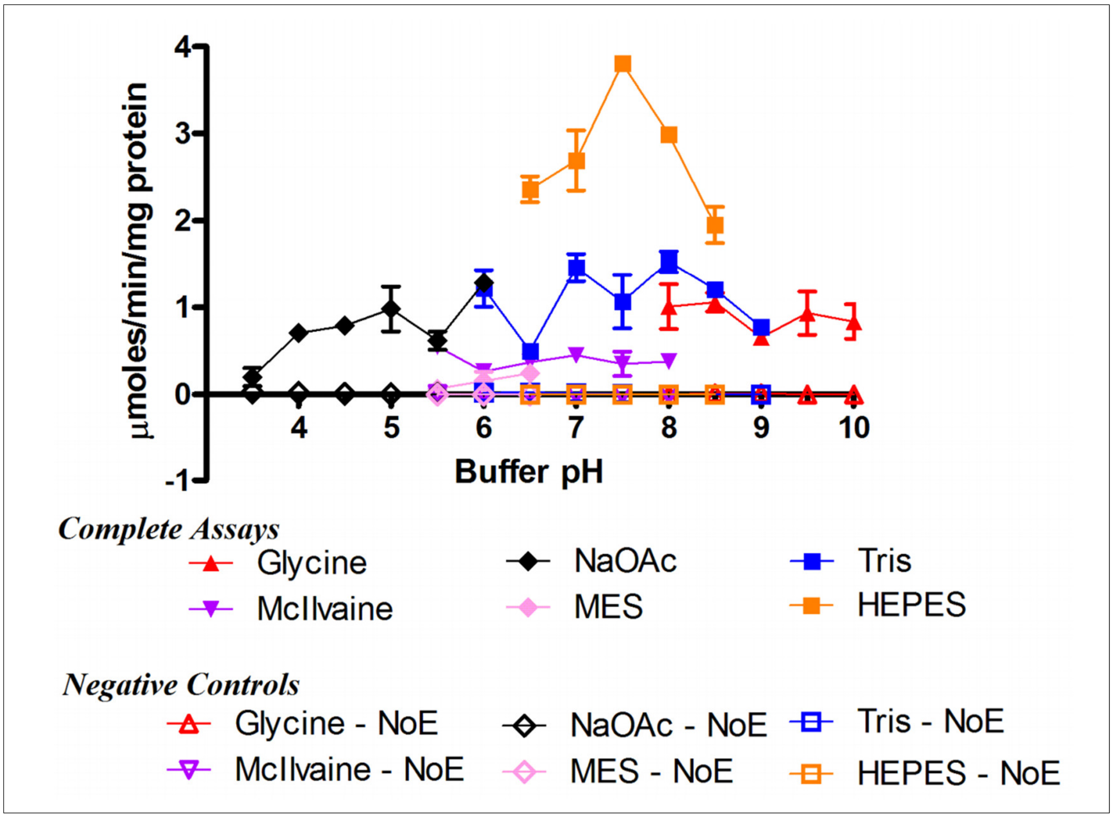

Figure S3. **Activity of recombinant Vv2KGR at various pH and in a range of buffer types**. Assays were performed with 0.4µg (final) purified recombinant enzyme, 40mM (final) 2KLG (substrate) and 0.25mM (final) NADH (coenzyme) at 37°C.

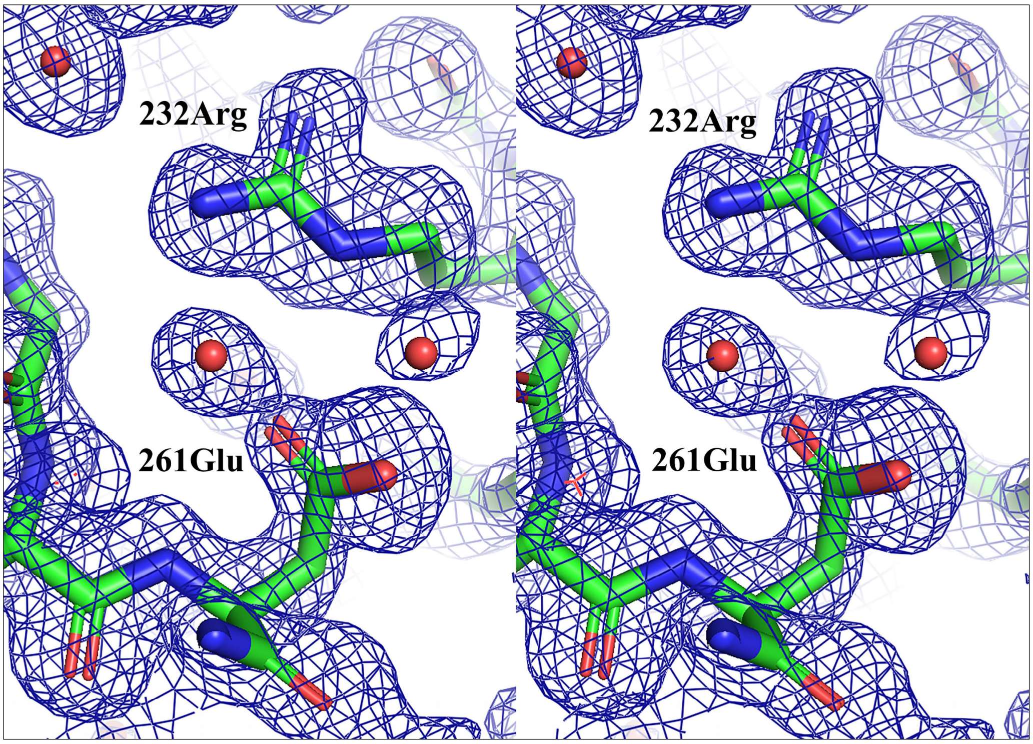

Figure S4. Reduced model bias electron density map of the Vv2KGR structure. Composite omit map (blue mesh) of active site of Vv2KGR (Green sticks colored by element) contoured at 1σ and depicted in wall eye stereo.

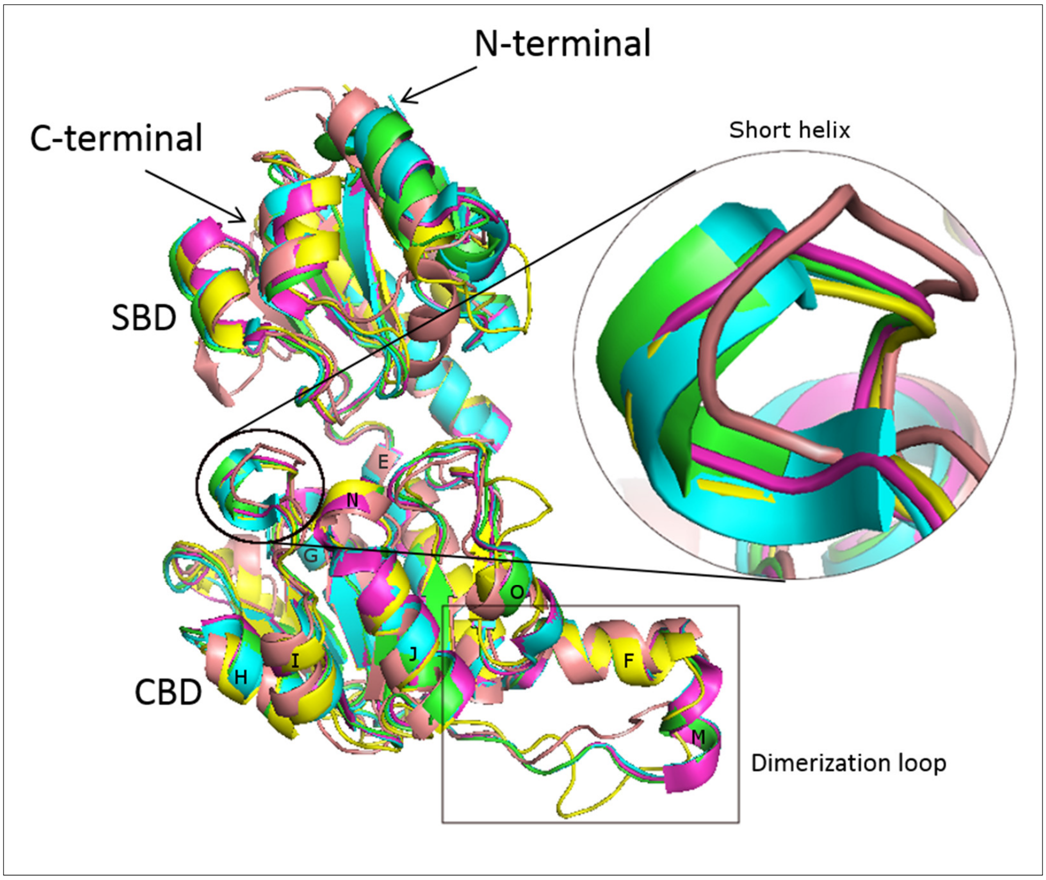

Figure S5. Structural superimposition of Vv2KGR (green) with other 2KDH proteins: apo-CbHPR (cyan), binary-CbHPR (magenta), GRHPR (yellow), Pt2GR (salmon). The helix index in the CBD domain was labelled according to Vv2KGR.

Table S1. Displays the pair-wise all atom RMSD value (Å) of the respective monomers and other 2KDH structures. The chain number and PDB IDs of Vv2KGR and 2KDHs were indicated accordingly.

		**Vv2KGR chain A**
VV2KGR chains	**B**	0.344
	**C**	0.273
	**D**	0.431
Hydroxypyruvate/glyoxylate reductase	**3BA1**	0.452
	**3BAZ**	0.500
	**4LSW**	1.575
	**2GCG**	1.203
	**2DBZ**	1.072
D-lactate dehydrogenase	**3WX0**	4.400
	**3WWZ**	1.883
	**3KB6**	1.380
	**4CUJ**	4.275

D-glycerate dehydrogenase	**1GDH**	3.347
Phosphateglycerate dehydrogenase	**2G76**	1.414
	**1YBA**	2.041
Transcription co-repression Ctbp dehydrogenase	**1HKU**	1.239
	**1MX3**	1.235
	**4U6S**	1.165
